# Recent Progress of the Preparation and Application of Electrospun Porous Nanofibers

**DOI:** 10.3390/polym15040921

**Published:** 2023-02-12

**Authors:** Pu Wang, He Lv, Xianyang Cao, Yanan Liu, Deng-Guang Yu

**Affiliations:** 1 School of Materials and Chemistry, University of Shanghai for Science and Technology, Shanghai 200093, China; 2 Higher Vocational and Technical College, Shanghai University of Engineering Science, Shanghai 200437, China

**Keywords:** electrospinning, polymer, porous fiber, air purification, water treatment

## Abstract

Electrospun porous nanofibers have gained a lot of interest recently in various fields because of their adjustable porous structure, high specific surface area, and large number of active sites, which can further enhance the performance of materials. This paper provides an overview of the common polymers, preparation, and applications of electrospun porous nanofibers. Firstly, the polymers commonly used to construct porous structures and the main pore-forming methods in porous nanofibers by electrospinning, namely the template method and phase separation method, are introduced. Secondly, recent applications of electrospun porous nanofibers in air purification, water treatment, energy storage, biomedicine, food packaging, sensor, sound and wave absorption, flame retardant, and heat insulation are reviewed. Finally, the challenges and possible research directions for the future study of electrospun porous nanofibers are discussed.

## 1. Introduction

**Figure 1:**
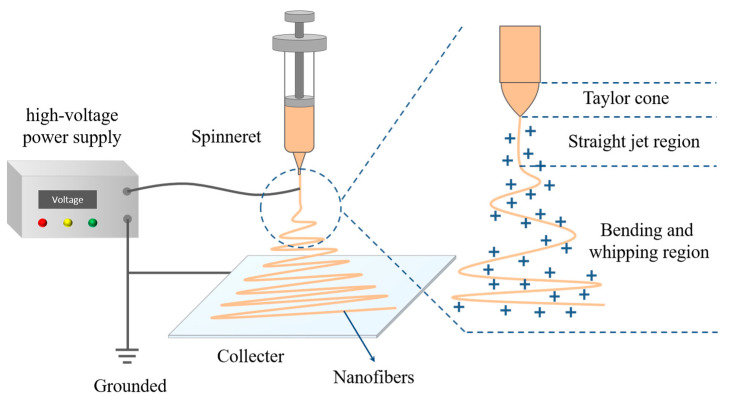
A process diagram for electrospinning.

Under the influence of external electrostatic fields, electrospinning technology, a sophisticated technique for fiber production, rapidly stretches polymers into continuous fibers with sizes varying from microns to nanometers [1,2,3]. Electrospinning technology has several advantages over other fiber film production techniques, including ease of operation, controllable process, high operability, low cost, and flexibility. Additionally, nanofiber membranes prepared by electrospinning also have special qualities, such as high porosity, large specific surface area, controllable fiber structure, and easy recovery. As shown in Figure 1, electrospinning equipment is primarily made up of components including a high-voltage power source, a propulsion pump, a syringe, a spinneret, and a collection apparatus [4,5,6]. The high voltage applied at the spinneret tip is used as the viscous electrospinning solution in the spinneret and is extruded at a steady rate to be charged. A polymer solution’s hemispherical surface is stretched into a cone, which is called a Taylor cone [7,8], when the applied voltage rises. The electrostatic force defeats the droplet’s surface tension once the voltage is sufficiently high. Charged solution jets are simultaneously released from the Taylor cone’s tip and divided into numerous smaller jets between the tip and the collection device. The jets are ejected from the surface of the liquid, forming straight jets, which subsequently enter the region of whipping and bending instability. The jets are rapidly stretched as a result of the whipping motions and then solidify into continuous fibers on the receiver with quick solvent evaporation [9].

A series of experimental variables, including the electrostatic field force [10,11], polymer parameters [12], solvent [13], spinneret [14,15,16], environment humidity [17], and temperature [18], have an impact on the morphology and structure of electrospun nanofibers. Coaxial and triaxial multifluid electrospinning processes have also been reported in recent years [19,20,21]. In this field, polymer fibers are functionalized by adding active ingredients in a mixed electrospinning process. Additionally, by adjusting post-treatment operations, different types of nanofibers, such as carbon nanofibers, metal oxide nanofibers, or composite nanofibers [22,23], can be produced (for instance, by heating nanofibers in different atmospheres). Electrospun nanofibers with various structures can be obtained using different spinning processes and by adjusting spinning parameters appropriately [24,25,26], such as porous fibers [27,28], hollow structures [29,30], hollow porous structures [31,32], core–shell fibers [33,34,35,36], Janus fibers [37,38], beaded fibers [39,40], and side-by-side structures [41,42].

Among them, porous nanofibers have drawn the most interest owing to their rich pore framework, large specific surface area, increased active sites, and easy functionalization, which show great potential in the areas of adsorption separation, water treatment, catalysis, energy storage, air filtration, drug delivery, tissue engineering, sensors, and food packaging [24,43,44,45,46].

In the process of electrospinning, fibers pile up layer by layer and form interconnected network pores. However, these pores are disordered, with large sizes and finite specific surface area, which cannot satisfy application needs to a large extent. The introduction of a porous structure can better solve these problems. Different from the pores between fibers, the porous structure of electrospun porous nanofibers refers to the presence of pores on the surface of a fiber or within a fiber, which can be divided into three categories: micropores (less than 2 nm), mesopores (about 2–50 nm), and macropores (more than 50 nm) [44]. Different pore sizes and shapes are needed for various applications. Due to the vast surface area of micropore structures with small pore diameters, some small molecules with small diameters can be adsorbed [47,48]. In addition, a single active center can also be stabilized by a unique spatial structure, making it available as a catalyst carrier. Mesoporous structures, which are larger than micropores, have uniform size, narrow distribution, and continuous adjustable apertures. Therefore, the ability of these mesoporous materials to adsorb and segregate some bigger molecules makes them crucial in the field of catalysis [49,50]. Macroporous structures with pores much larger than those of molecules frequently serve as spreading passageways. The porous structures are conducive to the diffusion and transfer of substances and are frequently employed in biomedical and battery energy materials [51,52,53,54]. In addition, a layered porous structure can integrate microporous, mesoporous, microporous, and other multilayer pores and can provide layered porous channels by designing the pore size and porous structure of materials at various scales, which can further improve the performance of materials and shows greater application prospects.

Combining the merits of nanofibers and porous structures, electrospun porous nanofibers have specific porosity and increased surface area, additional active sites, heterogeneous interfaces, and interior spaces, all of which are critical for the performance and function of nanomaterials. They can increase a material’s accessibility to an active site by accelerating the diffusion and transmission of materials. By creating nanofiber porous structures of various sizes, including the pores between, inside, and on the surface of fibers, it is possible to produce materials with various functions that can be used in various fields. We discuss the preparation and application of electrospun porous nanofibers in this review. Polymer materials and the pore-forming process of electrospun porous nanofibers are systematically introduced. Then, we summarize the most typical applications of electrospun porous nanofibers in air purification, water treatment, energy storage, biomedicine, tissue engineering, and other areas. Finally, the challenges and prospects of electrospun porous nanofibers are evaluated.

## 2. Polymers in Electrospun Porous Fibers

In the last few decades, researchers have made great efforts in electrospun porous fibers. Phase separation and sacrificial templates are widely used pore-forming methods for preparing electrospun porous nanofibers [55,56]. In the study of porous materials, polymers have the advantages of flexible chains, low cost, excellent spinnability, and easy processing and have been widely used as the main raw material. Porous nanofibers made from a variety of polymers have been successfully fabricated. The following list includes some common polymers used to create electrospun porous fibers (the specific pore-forming mechanism is described in Chapter 3).

Hydrophobic polymers are usually used to prepare electrospun porous nanofibers by the principle of phase separation. In electrospinning, for polymethyl methacrylate (PMMA), polylactide (PLA), polycarbosilane (PCS), and polystyrene (PS) polymers, chloroform (CF) and tetrahydrofuran (THF) usually serve as high-volatile solvents to form porous structures under high humidity by breath figures (BFs) [57,58]. Megelski et al. [59] prepared porous PS and PMMA nanofibers with CF as a solvent using electrospinning technology. Lu et al. [60] used THF as a solvent to prepare porous PS nanofibers with porous structures both on the surface of the fibers and within the fibers under high humidity. Although PS nanofibers exhibit porous structures by employing dimethylformamide (DMF) as a solvent, it only exists on the surface. Based on vapor-induced phase separation (VIPS) [61,62], porous nanofibers can also be prepared under certain humidity by using low-volatile solvents with good water miscibility, including DMF, dimethylsulfoxide (DMSO), and dimethylacetamide (DMAC) [63]. According to Wang et al. [64], porous PCS fibers could be created using DMF as a solvent.

In addition to BFs and VIPS, porous nanofibers can also be induced through nonsolvent-induced phase separation (NIPS) [65,66]. In NIPS, a solvent (capable of dissolving polymers)/non-solvent (immiscible with polymers) binary solvent combination can be selected for polymers such as polycaprolactone (PCL), PLA, polyvinylidene fluoride (PVDF), PS, polyacrylonitrile (PAN), CA (cellulose acetate), and so on. For example, porous PCL fibers were electrospun from a PCL/CF/DMSO solution [67]. In order to produce porous PLA nanofibers, a number of solvent/nonsolvent systems have been used, including dichloromethane (DCM)/hexane [68], DCM/DMF [69], DCM/DMAC [70,71], DCM/butanol [72], and CF/DMF [73]. In electrospun PVDF nanofibers, DMF/acetone solutions could be used to create porous fibers [74]. Both the surface and the interior of PS fibers could have porous structures by determining a suitable solvent ratio of THF/DMF [75]. H_2_O has also been employed as a nonsolvent to create porous structures in a PAN/DMF system [76,77,78]. Ji et al. [79] constructed porous CA fibers in a highly volatile binary solvent system of DCM/DMAC.

The sacrificial template method (removal of hydrophilic polymers by post-treatment washing) is usually used to prepare electrospun porous nanofibers for hydrophilic polymers, including polyethylene pyrrolidone (PVP), polyethylene glycol (PEG), and polyethylene oxide (PEO). Zheng et al. [80] reported that porous PVDF fibers can be obtained by removing PVP through washing in electrospun PVDF and PVP nanofiber membranes. With suitable solvents, some hydrophobic polymers, such as poly (AN-co-MMA) (PLLA) [81], PS [82], and poly (3-hydroxybutyric-3-hydroxyvaleric acid) (PHBV) [83], can also be extracted from precursors of immiscible polymer/polymer blends. In addition, a few polymers, including PMMA, PLA, PS, PEO, polyacrylic acid (PAA), polyvinyl alcohol (PVA), and polyvinyl butyral (PVB) are usually used as conventional precursor templates of porous electrospun nanofibers. These polymers degrade after high-temperature calcination in air and are removed from the matrix to obtain porous structures [84,85,86].

In short, porous fibers can be prepared with different solvents and post-processing methods depending on the various features of polymer materials. The resulting porous fibers have various pore states and morphologies. Additionally, different polymers are also used in different fields and can be selected according to the application.

## 3. Pore Formation in Electrospun Porous Fibers

Since porous nanofibers were effectively generated by electrospinning in the late 1990s, research has been focused on the preparation and optimization of these materials. After 20 years of research and development, numerous methods have been developed to construct the porous structures inside fibers in order to meet the requirements for various practical applications [87,88]. For porous nanofibers, either a polymer blend or a sacrificial component is electrospun and then post-treated to remove one of the polymers or the sacrificial component and to form a porous structure. Alternatively, pore sizes are directly generated by selecting an appropriate polymer–solvent system during electrospinning. The preparation methods for electrospun porous nanofibers are primarily split into the template method and the phase separation method based on various pore-forming mechanisms.

### 3.1. Template Method

A typical technique for creating porous nanofibers is the template method. There are two steps to the process: (1) a sacrificial template is added to the spinning solution to create a homogeneous solution and (2) the template is then removed by post-treatment (such as solvent extraction or heat treatment) to obtain porous nanofibers. Polymers, metals, metal oxides, and inorganic salts are frequently used as sacrificial templates in the production of porous structures [89,90,91,92,93,94,95,96,97,98,99,100,101,102,103,104,105,106,107,108,109]. This method has wide adaptability in the selection of polymer materials and can construct multifunctional porous structures in both hydrophilic and hydrophobic polymers.

#### 3.1.1. Using Polymers as Templates

**Figure 2:**
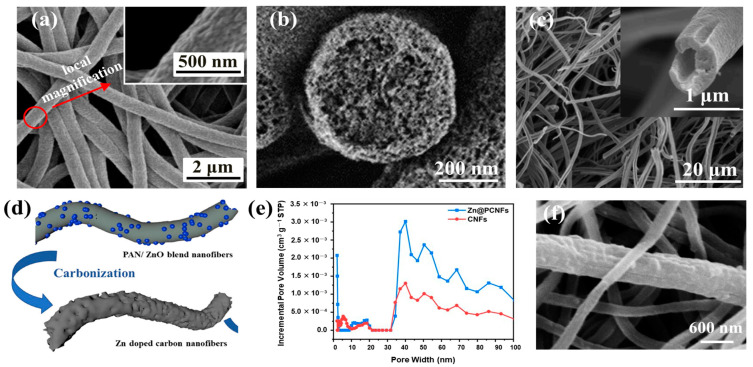
(**a**) SEM image of porous PAN nanofibers after amination. Reprinted with permission from Ref. [89], Copyright 2015, The Royal Society of Chemistry. (**b**) SEM image of cross-section of porous POM/PLLA nanofiber after chloroform immersion. Reprinted with permission from Ref. [92], Copyright 2016, The Royal Society of Chemistry. (**c**) SEM image of hollow, porous carbon nanofibers. Reprinted with permission from Ref. [31], Copyright 2021, Elsevier. (**d**) Schematic for the preparation of Zn@PCNFs and (**e**) pore size distribution of CNFs and Zn@PCNFs. Reprinted with permission from Ref. [97], Copyright 2022, American Chemical Society. (**f**) SEM image of porous PVDF/PAN nanofibers after HCl extraction. Reprinted with permission from Ref. [105], Copyright 2018, Springer.

In electrospinning, the polymer serves as a template to create porous structures in addition to acting as a matrix for nanofibers. To create porous nanofibers, a variety of mixed polymers with various characteristics can be employed. For example, hydrophilic polymers (such as PVP, PEO, and PEG) can be added to PAN, PVDF, and PCL nanofibers as sacrifice templates and can be further removed by immersion in deionized water. After drying, porous nanofibers can be formed. In Hong et al.’s [89] research, water was used as an extraction solution to remove PVP from PAN/PVP fibers, and diethylene-triamine (DETA) was then added to obtain aminated porous PAN fiber membranes (Figure 2a), which showed a relatively large adsorption capacity for lead ions. Ning et al. [90] obtained porous PVDF fibers coated with silver nanoparticles via removing PEO from a mixed solution of PVDF and PEO blends used as raw materials, which had effective methyl orange photocatalytic degradation activity. Gao et al. [91] prepared PCL/PEO fibers with different morphs by an electrospinning method. A porous PCL structure was formed by selectively removing the PEO phase in the fiber through water treatment. By varying the amount of PCL in the blend solution, porous nanofibers with different porosities and diameters were obtained. In addition to hydrophilic polymers, hydrophobic polymers can also be selectively eliminated from polymer mix precursors. Guan et al. [92] fabricated polyoxymethylene (POM)/PLLA nanofibers using electrospinning technology and then soaked them in chloroform for 12 h. PLLA was removed with the rapid evaporation of the solvent. At the same time, the fibers’ surfaces developed porous structures, and their interiors formed nanochannels (Figure 2b). The porous membrane showed a high oil absorption capacity.

Thermal treatment is an additional approach to generating porous structures in porous nanofibers. The polymer template is typically entirely decomposed by heating the polymer mix precursor to a specific temperature (usually the decomposition temperature of the sacrificial phase). Due to their different thermal stabilities, polymers with lower thermal stability are pyrolyzed into pores in the fibers during heat treatment, while polymers with higher thermal stability are carbonized into the fiber skeleton. The compatibility (or solubility) and thermal stability of different polymers can be used to influence the size and configuration of pores, and significant variances in these properties result in larger pores. Asare et al. [31] used PAN and PMMA as raw materials to prepare nanofibers by blending electrostatic spinning and coaxial electrostatic spinning. After high-temperature carbonization, PMMA was completely decomposed, forming a porous structure (Figure 2c). The fiber’s electrochemical performance was enhanced by its porous structure. In order to maintain nanofiber shape and skeleton, the sacrificial polymer’s breakdown temperature must be greater than that of the polymer matrix. In contrast to solvent removal methods, the integrity of porous fibers may be damaged after heat treatment, as heat flow usually inevitably causes damage to the original nanofibers.

#### 3.1.2. Using Metals or Metal Oxides as Templates

For the fabrication of porous fibers, some metals or metal oxides, such as SiO_2_ [93,94], Sn [95], ZnO [96,97], Fe_3_O_4_ [98], and Ni [99,100], are commonly used templates. By using suitable nanoparticles as templates, pores’ sizes and morphologies can be easily regulated.

Highly porous carbon nanofibers (PCNFs) were created by Nan et al. [94] utilizing electrospinning, carbonization treatment, and acid etching. The blend solution of polyamide acid (PAA) and tetraethoxysilane (TEOS) was first electrospun. The TEOS decomposed into SiO_2_ and evenly dispersed in the fibers after carbonization. To eliminate SiO_2_ nanoparticles, the fibers were finally immersed in an acidic solution called HF. The prepared PCNFs had a huge surface area and a microporous structure, with excellent lithium ion storage capacity (730 mAh/g). After 50 cycles, it still showed good reversible capacity (445 mAh/g). Nie et al. [97] obtained porous carbon nanofibers (Zn@PCNFs) containing zinc by adding ZnO nanoparticles to PAN nanofibers and then carbonizing them at a high temperature (Figure 2d). PAN was carbonized into a skeleton in the carbonization process, and some ZnO was reduced to Zn and further evaporated to produce pores. The produced fibers had greater surface areas and porosities than PAN carbon nanofibers (Figure 2e), which enhanced the adsorption capacity of methylene blue (MB). Fe_3_O_4_ nanoparticles were introduced by Liu et al. [98] to a PVA solution for electrospinning and then soaked in a glutaraldehyde (GA) and HCl acetone solution for 12 h. The Fe_3_O_4_ nanoparticle template was removed while the PVA was crosslinked, and crosslinked porous PVA nanofibers were obtained. The pores, which had an average size of 12 nm, were uniform throughout the nanofibers. Compared with pure fibers, the crosslinked porous PVA nanofibers showed higher thermal stability. The successful fabrication of porous PVA nanofibers provided a reference for preparing other porous fiber membranes using nano-Fe_3_O_4_ as template.

#### 3.1.3. Using Inorganic Salts as Templates

In addition to polymers and metal oxides, the production of porous electrospun nanofiber templates can also be performed using certain kinds of inorganic salts, such as CaCO_3_ [101,102,103], NaHCO_3_ [104,105], NaCl [106], GaCl_3_ [107], and dialkyl sodium sulfate (SDS) [108].

In Li et al.’s [109] research, PAN and PVP blends were employed as a solution for electrospinning, and then fibers were mixed with salt in an acid solution at a specified time and temperature using water baths with different concentrations of HCl to extract salt. In this case, nanoporous PAN microfibers with ultra-high specific surfaces were prepared by a two-step method. Mehraban et al. [102] used HCl to remove a CaCO_3_ template from PAN/CaCO_3_ nanofibers to produce porous nanofibers, and the porous fibers were also studied. Adhikari et al. [103] proposed a technique for creating TiO_2_ porous nanotubes utilizing PVAc as a matrix material and incorporating coaxial electrospinning with heat treatment and HCl etching. The dissolution of CaCO_3_ led to pore formation at its location. Compared with nonporous TiO_2_ nanofibers, the synthetic porous TiO_2_ nanotubes were more biocompatible for bone implantation and regeneration. Mokhtari-Shourijeh et al. [105] added NaHCO_3_ into a PVDF/PAN blend solution and prepared PVDF/PAN nanofibers using electrospinning technology. The fibers were immersed in a HCl solution to remove NaHCO_3_ and produce porous PVDF/PAN fibers (Figure 2f). The obtained porous structure increased the nanofibers’ surface areas, further improving the adsorption capacity of dyes.

In summary, the preparation of porous nanofibers by electrospinning can select different sacrificial substances as templates and obtain them through post-treatment. When using metals or metal oxides as templates, they need to be removed with acid, while polymers and inorganic salts as sacrificial templates can be simply removed by washing or carbonization. Therefore, the uses of polymers and inorganic salts as sacrificial components have better environmental friendliness and greater application prospects. A small amount of sacrificial phase may persist in the polymer matrix and alter the characteristics of nanofibers, even though the majority of the sacrificial phase can be eliminated from the precursor with suitable post-treatment. In addition, the process of constructing a porous structure is relatively complex and has strict requirements for the composition of the sacrificial phase and post-treatment.

### 3.2. Phase Separation Method

The phase separation mechanism is another method to prepare porous structures in the context of polymer nanofibers. Three categories are typically used to categorize the phase-separation-mechanism-based fabrication of porous nanofibers [110]: steam-induced phase separation (VIPS), thermally induced phase separation (TIPS), and nonsolvent-induced phase separation (NIPS). The electrospinning of porous nanofibers may include one or more phase separation techniques. The preparation of porous materials via phase separation methods requires suitable solvents and polymers.

#### 3.2.1. Steam-Induced Phase Separation (VIPS)

The following describes how VIPS produces electrospun porous nanofibers: (1) A hydrophobic polymer is immersed in a low-volatile solvent to build a homogeneous solution. (2) In the process of jetting, the low-volatile solvent evaporates slowly, and the water vapor in the air interacts with the surface of the fiber and easily diffuses inside the fiber, mixing with the solvent to form a nonsolvent of the polymer. (3) The polymer and solvent quickly separate into a polymer-rich phase and a solvent-rich phase. (4) Lastly, the polymer-rich phase eventually solidifies into the entire skeleton of the nanofiber, while the solvent-rich phase forms holes inside the fiber.

**Figure 3:**
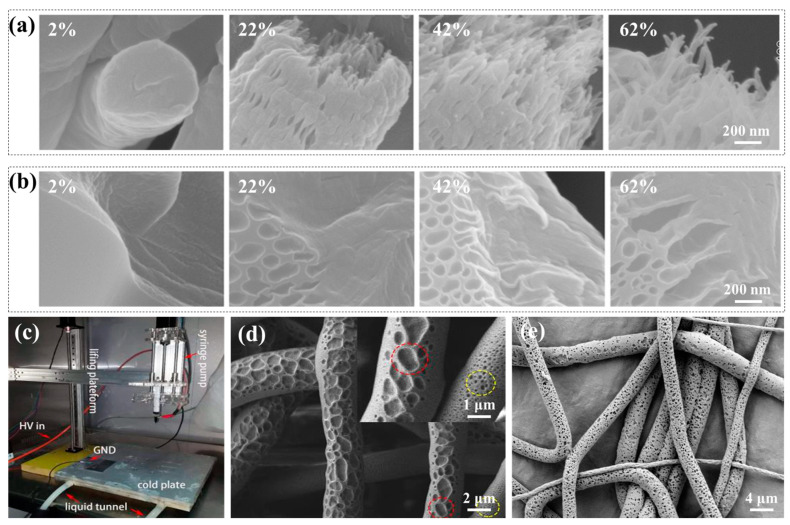
(**a**,**b**) SEM micrographs of 20 wt.% PS solution in DMF and THF under various relative humidities, respectively. Reprinted with permission from Ref. [60], Copyright 2013, American Chemical Society. (**c**) Configuration of a low-temperature electrospinning system; (**d**) SEM micrograph of PCL porous nanofibers. Reprinted with permission from Ref. [114], Copyright 2019, Elsevier. (**e**) SEM micrograph of porous PCL nanofibers fabricated in a solvent system of CF/DMSO. Reprinted with permission from Ref. [67], Copyright 2015, Elsevier.

The pore formation in VIPS is significantly influenced by the relative humidity of the air and the solvent’s volatility. In a DMF solvent system and a high-humidity environment, Pai et al. [111] attributed the generation of internal porosity of PS electrospun nanofibers to the miscible solubility of the solvent and water. They believed that, when water vapor saturated the area near the interface between the jet and air, liquid (DMF)–liquid (water) phase separation occurred rapidly, and the water in the air diffused and penetrated into the fibers. With the solidification of the fibers, the captured water formed pores. Lu et al. [60] used DMF as solvent in the process of electrospinning PS under different relative humidities. As shown in Figure 3a, they found that relative humidity influenced the fiber’s interior pore formation in addition to its surface shape. Low relative humidity prevents phase separation from occurring, resulting in the formation of smooth-faced nanofibers. Increasing the relative humidity can promote the accumulation and penetration of water vapor on the surface of a fiber, resulting in rapid phase separation and, thus, forming a porous structure. However, small circular depressions a left on a fiber’s surface after drying if the relative humidity is too high. The reason is that, while the solvent quickly evaporates, tiny water droplets adhere to the polymer fiber’s surface.

By choosing various volatile solvents, the porosity structure of a fiber can also be modified. When DMF with low volatility was used, Lu et al. [60] also found that pores were generated both on the fiber’s surface and inside in the preparation of electrospun PS nanofibers (Figure 3a). When THF with strong volatility was employed as the solvent, only a porous sheath layer was generated on the fiber’s surface, and the interior was typically solid (Figure 3b). This was related to the solvent’s volatility. When a solvent is less volatile, water molecules have enough time to enter the fiber before the polymer dries entirely, causing phase separation and the development of fiber holes. However, when a high-volatile solvent is applied, the interface between the air and the fiber is often saturated because of the presence of highly volatile solvent molecules, which prevents water molecules from infiltrating and phase separation from occurring. The quick evaporation of a solvent causes the nearby area to cool at the same time that water molecules condense on the surface of the fiber, leaving holes. As a result, the formation of pores on a fiber’s surface and inside it is greatly influenced by the volatility of solvents. By choosing an appropriate proportion of different solvent compositions, a porous structure can be produced on the surface and inside of a nanofiber simultaneously through the action of mutual competition. In addition, the morphology of porous fibers can also be impacted by the selection of different solvents, which is crucial for constructing hierarchically porous structures. Li et al. [112] reported the effects of various solvents (methylene chloride, acetone, CF, THF, and ethyl acetate) on the microstructure of a PMMA nanofiber and discussed its formation mechanism. Fibers with different pore morphologies were obtained in different solvents. When acetone (Ac) was used as the solvent, the fibers showed a bead structure. When choosing methylene chloride, CF, or ethyl acetate as the solvent, the fibers showed a banded structure. When THF was employed as the solvent, the fibers had a uniform structure, but the surface collapse was serious. With the exception of using acetone and THF, the fiber surfaces developed a porous structure. With a poor-volatile solvent (CF), elliptic pores were formed, while circular pores were with a high-volatile solvent (methylene chloride and ethyl acetate).

The VIPS method has high pore-forming efficiency, but the relative humidity and solvent volatility need to be strictly controlled. In addition, it is necessary to select hydrophobic polymers to generate porous nanofibers using VIPS in conjunction with electrospinning, and the solvent must be well miscible with water.

#### 3.2.2. Thermally Induced Phase Separation (TIPS)

The following steps describe the TIPS method used to fabricate electrospun porous nanofibers: (1) A polymer is dispersed in a poor-volatile solvent to generate a homogeneous solution. (2) The precursor solution is electrospun into fibers, and (3) phase separation between the polymer and the remaining low-volatile solvent in the fiber occurs during cooling because of the large temperature difference between the nanofiber and the nearby microenvironment. (4) Finally, porous structures are formed inside the nanofibers.

The temperature difference between nanofibers and the environment is the primary driver for TIPS. The creation of a porous structure is more advantageous when there is a greater temperature difference. Generally, there are two strategies to increase the temperature difference: one is to use a liquid bath at a very low temperature or to collect electrospun polymer nanofibers with a freezing collector, while the other is to increase the microenvironment temperature during electrospinning. McCann et al. [113] used electrospinning technology to fabricate fibers that were placed directly into a liquid nitrogen bath. Due to the extremely low temperature of the liquid nitrogen, the fibers cooled down quickly, and phase separation between the remaining solvent and the polymer occurred, resulting in the formation of a highly porous structure. Li et al. [114] prepared porous PCL nanofibers by designing a self-made vertical low-temperature electrospinning system that could temporarily solidify polymer jets on a freezer collector (Figure 3c). When the polymer jet temperature was –3.6 °C, TIPS transition occurred in a PCL/glacial acetic acid (GAC) solution, resulting in crystallization of the GAC. When the jet was sprayed on the frozen substrate at a specified distance of less than 110 mm, porous fibers appeared after vacuum freeze-drying (Figure 3d). The experiments showed that that the production of pores on the fibers throughout electrospinning required a freezing temperature and a significant amount of residual solvent. Ye et al. [115] successfully prepared isotactic polypropylene (iPP) fibers with a layered porous structure by high-temperature electrospinning technology combined with TIPS. By regulating the temperature of the polymer jet to 300 °C during electrospinning and collecting the nanofibers in a 25 °C revolving drum, the jet cooled rapidly. Within 0.35 s, there was rapid phase separation that resulted in a porous structure.

Membranes can be prepared from semicrystalline polymers that are insoluble in solvents at ambient temperatures using TIPS. In addition, TIPS processes are typically binary systems and require fewer variables to control than triadic NIPS systems. However, owing to the complicated circumstances of the entire process, pores created by various temperature differences have different morphologies and smaller pore sizes. Meanwhile, compared with other phase methods, the TIPS method is also relatively energy intensive.

#### 3.2.3. Nonsolvent-Induced Phase Separation (NIPS)

The preparation process of electrospun nanoporous fibers fabricated by NIPS is as follows: (1) A polymer/solvent/nonsolvent ternary solution is prepared by dispersing a polymer in a volatile solvent/low-volatile nonsolvent solution. (2) During the electrospinning jet process, solvent evaporation causes a shell to solidify rapidly, while the evaporation rate of the nonsolvent is slow, which causes it to permeate and diffuse into the jet. (3) The composition of the solution system changes rapidly, and phase separation occurs, forming a nonsolvent and polymer two-phase heterogeneous binary solution. (4) The nonsolvent volatilizes at a slower rate and remains in the polymer system. After the nonsolvent is completely volatilized, it leaves pores in its position, forming a porous structure. The earliest nonsolvent induction method for pore formation was to dissolve a polymer in a two-component solvent solution combination made up of a non-volatile bad solvent and a volatile good solvent and then to cast the two-component solution onto a glass substrate. The polymer accumulated in the solution contained the poor, non-volatile solvent when the excellent solvent volatilized and, finally, solidified to form a porous membrane structure [116].

In the process of preparing porous materials by NIPS, sufficient interaction time between the nonsolvent and the polymeric fluid jet is a key factor to promote the creation of interior pores. Therefore, by designing suitable solvent/nonsolvent combinations, the differences in volatility between the solvent and nonsolvent, as well as the sizes and morphologies of holes, can be precisely regulated. Qi et al. [72] prepared porous PLA electrospun nanofibers using electrospinning technology and NIPS. DCM, as a solvent of PLA, had high volatility, while DMF, as a nonsolvent, had low volatility. The significant difference between the solvent and nonsolvent volatility led to rapid volatilization of DCM and a slower volatilization rate of DMF in the process of electrospinning, resulting in liquid–liquid separation and the production of porous structures inside the fiber and on the surface of the fiber. If the difference between the solvent and nonsolvent volatilization rates is not sufficient, the speed of solution entry into the two-phase zone is slowed, making it difficult to form pores.

The porosity structure of a fiber can be modified by adjusting the solvent/nonsolvent component ratio to some extent. To create porous PS nanofibers, Lin et al. [75] used DMF as a solvent and employed THF as a nonsolvent. The generation of porous structures on the fiber’s surface and inside was observed to increase with the concentration of DMF in the DMF/THF binary solvent. This was due to the mutual diffusion of the solvents in the jet and the competition between the fast phase separation and the crystallization of the surrounding water. In contrast, pores could only be generated on a fiber surface when the content of THF was high. The rapid phase separation brought on by solvent evaporation and the following cooling and curing of the fiber were what caused pores to form on the surface. Katsogiannis et al. [67] used the NIPS mechanism to prepare porous PCL fibers and studied how solvent characteristics affected the size and shape of PCL nanofibers. As shown in Figure 3e, on the fiber surface, pores were made using a solution of CF (a good solvent) and DMSO (a bad solvent). They found that, when 12.5% (*w*/*v*) PCL was dissolved in a CF/DMSO (*v*/*v*: 75–90%) solution, porous, beadless nanofibers with approximate diameters in the range of 1470–2270 nm could be obtained. Therefore, a high solvent/nonsolvent ratio was favorable for pore formation. In addition to a polymer solvent, water can be introduced straight to a polymer solution as a nonsolvent to make porous nanofibers. In the study of Yu et al. [77], a PAN/DMF/H_2_O solution was employed to fabricate electrospun porous nanofibers. In order to cause phase separation and create porous structures, water was utilized as a nonsolvent. With increases in the water content and polymer concentration, fibers’ specific surface areas and diameters increased.

Alternatively, a polymer solution can be electrospun directly into a nonsolvent cell, where fibers are collected before the solvent evaporates completely to obtain a porous structure. Nayani et al. [117] used the electrospinning technique to fabricate porous PAN fibers by spinning a PAN/DMF solution into a nonsolvent cell. When fibers made contact with the surface of a nonsolvent bath, phase separation occurred, and pores were generated. They also found that the miscibility of the solvent and nonsolvent also had a significant effect on the shape of nanofibers. By spinning into an ethanol or water bath, fibers having pores on the surface and interior could be generated, while no pores were formed by spinning into a hexane bath. The reason is that DMF is highly miscible with water or ethanol and, thus, could easily penetrate into the interior of PAN fibers. In this case, liquid–liquid separation took place, resulting in the generation of structures with high porosity. Therefore, selecting an appropriate solvent and nonsolvent system is crucial for the porous structure resulting from NIPS.

Compared with the other two phase separation methods, the pores created by NIPS are irrespective of outside humidity. The pores are elliptical and typically smaller in size. Nanofibers with both surface and interior porous structures can be obtained in one step by selecting an appropriate solvent-to-nonsolvent ratio and polymer content. NIPS typically requires two or more solvents. The formation mechanisms, formation conditions, and pores formed by different phase separation methods are shown in Table 1.

## 4. Applications of Electrospun Porous Nanofibers

Owing to their large surface area and rich pore structure, highly porous nanofibers based on electrospinning have gained a great deal of attention in the past few decades. In the environmental field, a porous structure can improve specific surface area and give membranes a superhydrophobic surface, as well as being employed as a trap for filtering. In terms of energy storage, high porosity and large specific surface area are advantageous for maximizing the use of active sites during redox reactions and high-flux mass transfer [122]. In the field of biology, porous structures can improve drug release, further enhancing drug efficacy, and can improve cell adhesion and have a great deal of promise for vascular tissue engineering and regeneration [123]. Based on the developed electrospun porous nanofiber preparation process, researchers can take advantage of the characteristics of porous nanofibers in combination with other processes, such as fiber surface modification [124,125] or the addition of functional substances [126,127,128], which can have a significant impact in different fields. Electrospun porous nanofibers are employed extensively in a variety of disciplines, including air purification, water treatment, energy storage, biomedicine, tissue engineering, food, sensors, and so on.

### 4.1. Air Purification

#### 4.1.1. Air Filtration

Air pollution has attracted more and more attention in recent years. It can result in major health issues, such allergies, cardiovascular disease, and respiratory illnesses, all of which are mainly attributed to particulate pollutants smaller than 2.5 μm in diameter suspended in the air. Therefore, capturing and filtering these harmful, fine particles is an effective way to protect oneself from air pollution. In order to effectively capture particles and enable airflow to pass through easily, an optimal electrospun air filter ought to possess a high porosity and a small fiber diameter. Electrospun porous nanofibers have a higher surface area and a porous structure with interpore connectivity, which can effectively collect tiny particles [129,130].

**Figure 4:**
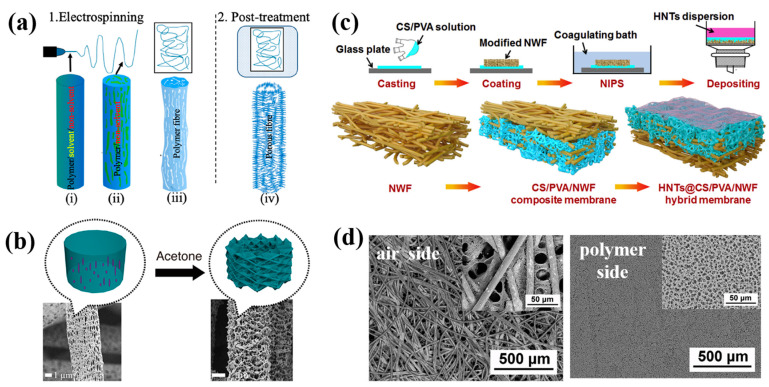
(**a**) Schematic diagram of the preparation of porous PLLA nanofibers; (**b**) SEM photos of nanofibers before and after acetone post-treatment. Reprinted with permission from Ref. [69], Copyright 2019, American Chemical Society. (**c**) Schematic illustration of the fabrication of HNTs@CS/PVA/NWF nanofibers; (**d**) SEM photos of CS/PVA/NWF air filtration membranes. Reprinted with permission from Ref. [131], Copyright 2020, Elsevier.

By using electrospinning technique, Li et al. [70] produced eco-friendly polylactic acid (PLA)/chitosan (CS) porous nanofibers. The filtration capacity and antibacterial properties of pollutants in the air were tested. The results showed that the microstructure of the porous fibers was significantly affected by the concentration of the electrospinning solution. When the concentrations of polylactic acid and chitosan were 8% and 2%, respectively, uniform, smooth porous nanofibers could be obtained. At relatively high humidity, the larger the pore size of the fiber, the higher the porosity. When the optimum mass ratio of CS to PLA was 2.5:8 and the rate of air flow was approximately 14 cm/s, the porous membrane possessed the best filtration performance. The pressure drop reached 147.6 Pa, and 98.99% of the filtration was successful. The antibacterial experiments demonstrated that the porous membrane also had a good bacteriostatic effect on Staphylococcus aureus and Escherichia coli. The porous fiber membrane might be employed as an air filtration material with outstanding purification capability, according to air purification experiments. Song et al. [69] dissolved PLLA particles in a DCM and DMF (19:1 *w*/*w*) dual-solvent system for electrospinning to obtain PLLA nanofibers and then successfully prepared a layered porous PLLA membrane by an acetone post-treatment process (Figure 4a). Based on the theory of solvent-induced recrystallization, NIPS during electrostatic spinning and solvent-induced recrystallization in post-treatment were both thought to contribute to the production of the layered porous structure. The interior of the single fiber was divided into thinner fibers by the pores during NIPS. The acetone treatment expanded the PLLA fiber and encouraged the polymer chain to recrystallize, creating a layered porous structure (Figure 4b). Benefiting from their lager porosity and specific surface area, the produced porous PLLA fibers exhibited a low pressure drop and a superior filtering effectiveness for NaCl aerosol. The layered porous nanofibers with excellent surface area could have a wide range of potential applications in the air filtration sector.

In order to achieve higher air-filtering effectiveness and a lower pressure drop, Wang et al. [131] successfully fabricated an environmentally friendly air filtration membrane of alloxite nanotubes (HNTs)@Chitosan (CA)/polyvinyl alcohol (PVA)/nonwoven fabric (NWF) with a homogeneous, layered porous structure using dip coating, NIPS, and an HNT surface deposition method (Figure 4c,d). The hydrophilic modification of polyester NWF by a TiO_2_/H_2_O_2_ photocatalytic method was studied. The dip-coating NIPS method combined with NWF inhibited the transverse and longitudinal shrinkage of the fiber film during NIPS, greatly reduced the pressure drop, and also enhanced the mechanical properties of the nanofiber membranes. The surface deposition of HNT further reduced the pore size of the mixed film, which was conducive to the direct interception of larger bis(2-ethyl-hexyl) sebacate (DEHS) particles. In addition, the filtering effectiveness of DEHS particle removal was greatly improved by electrostatic interactions, hydrogen bonds within CS/PVA molecule chains, dipole–dipole interactions among CS/PVA/NWF and DEHS, and the diffusion adsorption of HNTs. The HNTs@CS/PVA/NWF film had a filtering effectiveness of 96.8% and a pressure drop of 143.9 Pa at a flow rate of 5.3 cm/s. Additionally, it also performed wonderfully in its antibacterial properties.

#### 4.1.2. Air Adsorption Separation

In recent years, the globe has been gradually warming due to large amounts of greenhouse gas emission. Carbon dioxide (CO_2_) has made a sizable contribution to global warming, and the release of CO_2_ into the atmosphere is increasing as a result of the burning of fossil fuels. Therefore, it is imperative to develop CO_2_-capturing technology and take the necessary measures to control the concentration of CO_2_ in the air. Traditional CO_2_-adsorbent materials show limited efficiency and low stability [132], while new, porous carbon nanofibers have great advantages in CO_2_ capture because of their adjustable pore structures, easy functionalization, huge surface areas, and excellent thermal, chemical, and mechanical stability.

**Figure 5:**
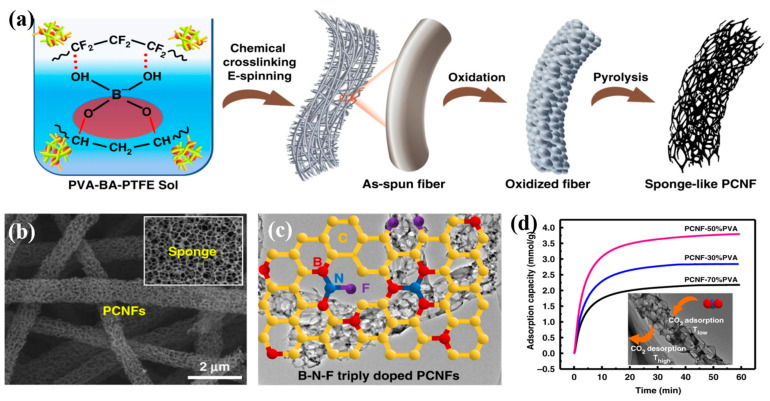
(**a**) Schematic diagram of PCNF synthesis via electrospinning and post-heat treatment; (**b**) SEM image of PCNFs; (**c**) chemical model of B-N-F triple-doped PCNFs; (**d**) CO_2_ adsorption performance of PCNFs. Reprinted with permission from Ref. [134], Copyright 2019, Springer Nature.

In order to create porous PAN nanofibers, Zainab et al. [133] employed a PAN and PVP blended solution as the electrospinning raw material and then washed and removed the PVP. After carbonization, porous/opening carbon nanofibers (PCNFs) with small diameters were formed. The CO_2_ adsorption capacity of the film was 3.11 mmol/g, about 20 times that of N_2_ (0.15 mmol/g), showing excellent CO_2_ gas selectivity and good trapping capacity. This was primarily because of the formation of a rough micropore structure after PVP washing and the formation of large pore sizes and opening pores in the fiber after carbonization treatment. More CO_2_ gas was absorbed by the PCNF nanofibers as a result of both of these aspects. After adsorption and desorption for 50 cycles, PCNFs still possessed high adsorption capacity and stability. Yan et al. [134] created PCNFs connected with medium and large pores for carbon dioxide adsorption by chemical crosslinking electrospinning and post-heat treatment (Figure 5a). Using polyvinyl alcohol (PVA) as a carbon precursor and boric acid (BA) and tetrafluoroethylene (PTFE) as a pore inducer and a crosslinking agent, respectively, after electrostatic spinning, the obtained fibers underwent oxidative dehydrogenation at 280 °C and N_2_ pyrolysis at 800–1200 °C. In this process, micro- and macrophase separation occurred, forming mesoporous and macroporous structures (Figure 5b), which later led to the development of B-F-N triple-doped PCNFs (Figure 5c). The obtained PCNF pores were evenly distributed with a maximum surface area of 750.6 m^2^/g and a porosity of 80%. The rapid cage control of CO_2_ molecules was facilitated by the big pores’ huge internal surface areas. The strong surface interaction between mesoporous and microporous structures promoted the adsorption of CO_2_. The maximum absorption rate was 3.9 mmol/g (Figure 5d), which proved that the high efficiency of both the adsorption and diffusion of PCNFs could improve CO_2_ capture.

In addition to CO_2_ adsorption, electrospun porous nanofibers can also effectively absorb volatile organic compounds (VOCs). Highly hazardous and carcinogenic VOCs are organic compounds with a low point of boiling and high steam pressure. VOCs are recognized as one of the most important environmental hazards. The adsorption method is one of the VOC treatment technologies that is effective and affordable and has the promise of recovery and reuse. Liu et al. [135] successfully prepared flexible PAN fibers with a large surface area and functionalized SiO_2_ aerogel by using electrospinning technology for the adsorption of VOCs. The specific surface area and porosity of the membrane were significantly increased by the honeycomb porous structure of the SiO_2_ aerogel. Under the same experimental conditions, PAN with 100% SiO_2_ nanofibers had the maximum adsorption capacity of 1841.1 mg/g for VOCs. Due to the storage of the largest SiO_2_ aerogel, the composite membrane had good surface area, acceptable pore size, and the largest pore volume and porosity. Si-O, Si-OH, and C=N organic functional groups also provided more active sites for VOCs. Therefore, the newly added porous structure could significantly improve the adsorption properties of the membrane material. Liang et al. [136] used polymer PAN and zeolitic imidazolate framework-8 (ZIF-8) hybrid nanofibers as precursors to prepare multilayer porous ZIF-8/PAN nanofibers via a calcination technique. Layered pores and numerous active sites that contained nitrogen were introduced into the fibers to promote the adsorption ability of the nanofibers. As a result, the adsorption capacity of benzene was increased to 694 mg/g. This exploration provided a certain reference value for the preparation of polymer/metal–organic framework (MOF)-derived nanofibers by electrospinning.

### 4.2. Water Treatment

As we all know, the most significant substance on earth is water. In recent years, water pollution has been one of the biggest global concerns to public health and a significant hazard to human health. Electrospun porous nanofibers have the advantages of rich pore structure, adjustable pore size, customizable channel interface, and easy functionalization. They show great potential in the filtration, adsorption, separation, and photocatalytic removal of pollutants in water.

#### 4.2.1. Membrane Filtration

Depending on screening effects, membrane filtration could effectively exclude materials of a particular size. However, due to high film thickness (more than 100 μm), partly closed pore channels, and low porosity (less than 80%), existing membranes have low permeability flux (usually less than 1000 L/m^2^/h) and large operating pressure (more than 100 KPa). Electrospun porous fiber membranes have large flux velocity and low membrane pressure in membrane filtration technology because of their interlinked open porous structure, high porosity, and customizable thickness [137], thus becoming the most potentially attractive filter.

**Figure 6:**
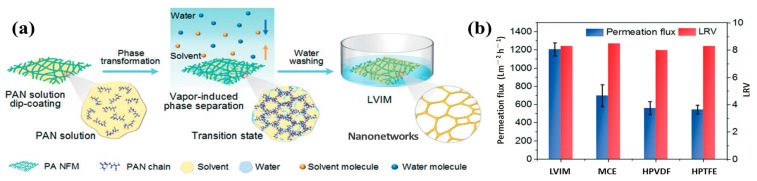
(**a**) Schematic diagram of the preparation procedure of porous microfiltration membranes; (**b**) bacterial removal efficiency of porous microfiltration membranes and commercial membranes. Reprinted with permission from Ref. [119], Copyright 2020, The Royal Society of Chemistry.

Tang et al. [119] prepared nanofiber/nanomesh composite porous microfiltration membranes for the interception of water-borne pollutants, including TiO_2_ particles and Escherichia coli, using electrospinning technology combined with VIPS (Figure 6a). Through the precise control of the solvent and relative humidity conditions, the prepared filter membrane had a submicron pore size of 0.19 μm, a high porosity of 93.2%, an ultra-thin thickness of 700 nm, and excellent interconnectivity. At a pressure of 5 kPa, the permeation flux of the membrane could reach 3907 L m^−2^ h^−1^, and the filtration ability could reach 99.75%. Additionally, according to Figure 6b, the membrane had greater filtration efficiency than the current commercial sterile membrane. The successful construction of this multifunctional membrane could provide inspiration for the design of high-quality films for numerous filtration applications. Chen et al. [138] used a poly(tetrafluoroethylene-co-hexafluoropropylene) (FEP)/polyvinyl alcohol (PVA) blend solution for electrospinning to prepare fiber film and then sintered it under a N_2_ atmosphere to obtain microfiber FEP porous films. When the weight ratio of PVA/FEP was 1:6, the sintering temperature reached 300 °C, and the sintering time was 10 min, the porous membrane showed strong hydrophobicity, a tiny pore diameter, and high porosity, which met the reasonable pore size of the filter membrane. The porous film also showed good mechanical properties. The porous membrane permeation flux could reach up to 15.1 L m^−2^ h^−1^ at a feed temperature of 80 °C and a transmembrane pressure of 0.06 MPa. The salt removal rate reached 97.99% when the feed NaCl content was 3.5 weight percent, which showed a broad range of application potentiality in the purification of seawater treatments.

#### 4.2.2. Oil–Water Separation

The rapid expansion of energy and chemical products has made the discharge of oily sewage increase, and toxic compounds have caused serious pollution to the ecosystem, even endangering human life and health. Therefore, it is imperative to remove significant quantities of oil from polluted water. Designing oil absorbents with large surface areas and proper hydrophobicity is important to increase the adsorption capacity and rate of oil in oily wastewater. Electrospun porous nanofibers are thought to be a viable candidate material for oil–water separation because they offer a high surface area and changeable wettability, which can increase oil adsorption properties [139,140].

**Figure 7:**
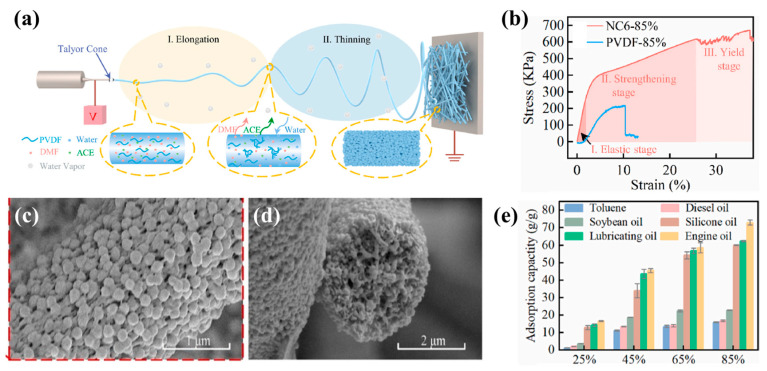
(**a**) Schematic diagram of electrospinning preparation of porous PVDF/CNC nanospheres; (**b**) stress–strain curve of porous PVDF/CNC and pure PVDF membranes; (**c**,**d**) SEM images of electrospun porous nanofibers with relative humidity of 85%; (**e**) adsorption capacities of porous PVDF/CNC membrane to different oils in different humidity. Reprinted with permission from Ref. [142], Copyright 2022, Elsevier.

By electrospinning crosslinked polyvinyl alcohol/nanoparticles (CPVANPs), Pane et al. [141] reported a strategy to fabricate a flexible porous nanofiber film (PVANP). On the basis of amino-functionalized hypercrosslinked polymer nanoparticles (AHCPNPs), a PVA/CPVANP membrane was prepared via electrospinning and was subsequently submerged in a para-phenylenediamine solution for 6 h. The porous membrane had excellent mechanical and tensile properties due to the good combination of rigid nanoparticles and large-molecular-weight, flexible PVA fibers. The high content of AHCPNPs provided the membrane with good oil capture performance, which was primarily due to the swelling and solvation actions of the three-dimensional crosslinked network provided by AHCPNPs. The microporous structure of the CPVANP film promoted the rejection of large oil droplets and brought high emulsified oil separation efficiency and large water flux to the film, thus showing high oil absorption capacity and a high-efficiency separation ratio of emulsified oil. Yan et al. [142] prepared a reusable polyvinylidene fluoride/nanocellulose (PVDF/CNC) porous fiber composite by one-step electrospinning (Figure 7a). The addition of CNC greatly increased the mechanical strength of the nanofiber film, which was 3.14 times that of pure PVDF (Figure 7b). In addition, the CNC’s surface had a lot of hydroxyl groups, which encouraged NIPS during the spinning process. A significant number of nanospheres were produced on the fiber’s surface, and interconnected pores are produced internally by varying the relative humidity (Figure 7c,d). The composite membrane possessed a large surface area and outstanding adsorption ability for oils (the maximum adsorption capacity of engine oil was 73.04 g/g) (Figure 7e). The NC6-85% composite’s adsorption retention rate for toluene was 85% after ten cycles. By adding reinforcing elements, the mechanical properties of porous fibers membrane can be increased. The complementary properties of different materials are beneficial to decrease a material’s application restrictions.

#### 4.2.3. Adsorption

The adsorption method has been proved to be one of the most economical ways to remove pollutants from polluted water due to its simple operation and small energy usage. The huge specific surface area of porous nanofibers produced by electrospinning can provide considerable adsorption capacity, so they can be used to absorb heavy metal ions, dyes, antibiotics, and other pollutants in water. Their porous structure can expand the exposed area of functional particles and provide more active sites. In addition, the interconnecting pores in a fiber can also promote the diffusion rate of pollutant molecules, thus improving the adsorption performance.

**Figure 8:**
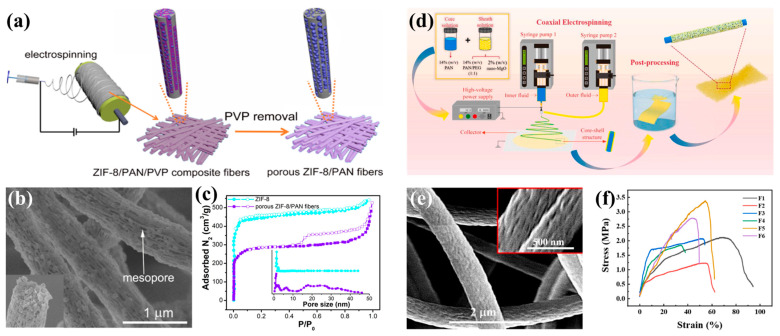
(**a**) Schematic diagram of the fabrication of porous ZIF-8/PAN nanofibers; (**b**) SEM photos of porous ZIF-8/PAN nanofibers; (**c**) N_2_ adsorption–desorption isotherm of porous ZIF-8/PAN nanofibers. Reprinted with permission from Ref. [144], Copyright 2021, American Chemical Society. (**d**) Synthesis pathways of porous PAN nanofibers loaded with nano-MgO functional particles; (**e**) SEM image of porous PAN nanofibers after post-treatment in deionized water (without nano-MgO); (**f**) comparison of mechanical properties of membranes (F1 and F2 are pure PAN and PAN with 2% MgO nanofibers, respectively, created by blended electrospinning; F3 and F4 are PAN@PAN with PEG and PAN@PAN with PEG and 2% MgO nanofibers, respectively, created by coaxial electrospinning; F5 and F6 are porous nanofibers obtained by post-processing PEG removal on the basis of F3 and F4, respectively). Reprinted with permission from Ref. [146], Copyright 2022, Elsevier.

Xu et al. [143] used PVP as a porogen to prepare polyethyleneimine (PEI)-porous polyacrylonitrile (PPAN) nanofibers (PPAN-PEI) via an electrospinning method, which were used to adsorb and remove methyl orange (MO) organic dyes from water. The obtained fiber film had an abundant porous structure, and the highly exposed pores enhanced the surface area in contact with the pollutants, providing a large amount of MO adsorption sites. In addition, through PEI modification, the surfaces of nanofibers loaded with rich amine groups not only improved the hydrophilicity but also enhanced the electrostatic attraction between the nanofiber film and methyl orange, with an adsorption capacity of methyl orange up to 726.45 mg/g, which was five times that of the adsorption–desorption cycle experiments, and the dye still maintained a high adsorption efficiency of 86.7%. It had a good reusable performance. By adjusting the size of the porous structures in the fiber, pollutant molecules of different sizes could be absorbed. Using PAN as a matrix and PVP as a porogen, Zhao et al. [144] prepared interconnected mesoporous porous zeolite imidazole framework-8 (ZIF-8)/polyacrylonitrile (PAN) nanofiber membranes utilizing an electrospinning method (Figure 8a). The SEM results showed that mesoporous structures were formed on the fiber surface during PVP removal, and monodispersed ZIF-8 particles were found embedded in these structures (Figure 8b). A N_2_ adsorption–desorption experiment also supported the mesoporous characteristics of the surface of ZIF-8/PAN nanofibers (Figure 8c). The presence of these mesoporous formed MOF–polymer interfaces inside the fibers not only accelerated the diffusion of pollutant molecules to the fibers, but also exposed more ZIF-8 adsorption sites, greatly improving the adsorption rate and capacity. The highest adsorption capacity of the composite membrane for TC reached 885.24 mg g^–1^. Therefore, it is of great significance to construct interconnected mesoporous structures for the adsorption of macromolecular pollutants in wastewater. Dou et al. [145] prepared a novel, ultra-porous polyimide hollow carbon nanofiber membrane (CNFM) as a ciprofloxacin (CIP) adsorbent by a blending electrospinning technology. The composite fibers were directly electrospun from a solution of polyamide acid (PAA) and methyl methacrylate (PMMA), heated to 1000 °C in a tube furnace at 5 °C/min, and carbonized in a N_2_ atmosphere for 3 h to obtain porous CNFM. The differing thermal stabilities of the PAA and PMMA polymers were thought to be the reason for the microphase separation of blends, resulting in the creation of hollow structures in the nanofibers, which could significantly reduce the adsorption time and increase the adsorption efficiency. The specific surface area of the adsorbent was 2327 m^2^/g, and the pore volume was 1.26 cm^3^/g. The adsorbent had a significant adsorption effect on CIP. In the adsorption process, hydrophobic interactions and pore filling both played important roles.

In order to improve the mechanical properties of porous fibers, Xu et al. [146] designed porous core–shell nanofibers with PAN as the core and MgO supported by PAN/PEG as the shell by coaxial electrospinning (Figure 8d). Through post-treatment, the PEG was eliminated, and a porous structure was introduced on the surface of the fiber (Figure 8e), expanding the exposure area of functional particles and improving the adsorption performance. The maximum adsorption capacity of the porous membrane for Cu^+^ was 354 mg/g. In addition, the mechanical properties of the membrane were tested, indicating that the core layer of PAN could provide favorable support and significantly enhanced the fiber’s mechanical properties (Figure 8f). As a result, adding a core–shell structure to porous nanofiber membrane preparation could alleviate damage in porous structure to the mechanical properties of nanofibers.

#### 4.2.4. Photocatalysis

As promising catalysts, electrospun porous nanofibers also exhibit excellent application potential in the photocatalytic treatment of pollutants in water. Porous structures can not only increase the adhesion points of catalysts in the fibers and provide more active sites, but can also enhance light penetration and reduce diffusion resistance, thus accelerating mass transfer [147,148]. Eventually, the photocatalytic activity of the degradation of organic contaminants in wastewater is improved.

Qian et al. [149] fabricated graphite carbon nitride (g-C_3_N_4_)/polylactic acid (PLA) nanofiber composite materials with layered mesoporous and macroporous structures by centrifugal electrospinning and alkali treatment for the photocatalytic degradation of carbazepine. A g-C_3_N_4_ photocatalyst was dissolved in a PLA (solvent: DCM/DMAC) solution for electrospinning, and the obtained film was then submerged in a NaOH solution for 15 min. The phase separation and the presence of g-C_3_N_4_ nanosheets led to a porous structure on the fiber’s surface and inside. With the NaOH soaking treatment, hydrolyzed ester bonding of PLA improved the porous structure and enhanced the pore size, which avoided the wrapping of g-C_3_N_4_ and increased the exposure of the catalyst. Under the same experimental conditions, porous g-C_3_N_4_/PLA nanofibers provided more active sites, not only promoting contact between catalysts and pollutants, but also providing more electron and hole utilization, and the photocatalytic property was superior to that of nonporous g-C_3_N_4_/PLA nanofibers. Consequently, a porous structure is crucial in the development of efficient photocatalytic nanofiber materials. It is beneficial to promoting the photocatalytic performance of a fiber membrane by introducing porous structure into the fiber to construct abundant porous channels.

**Figure 9:**
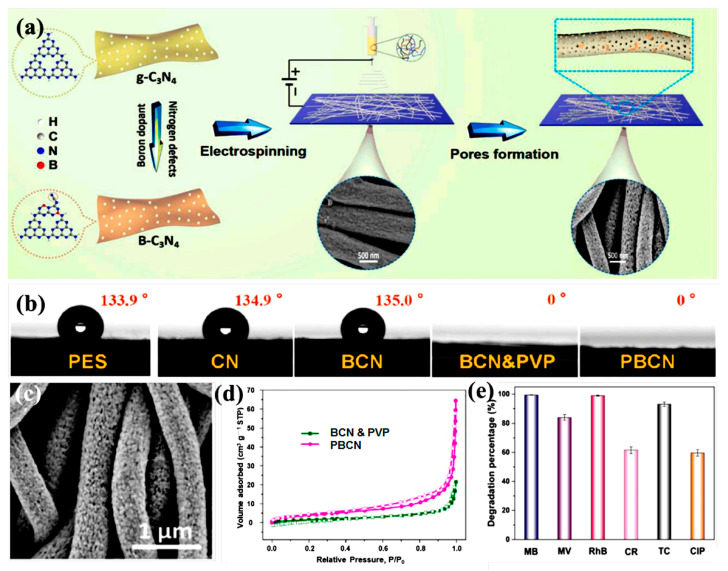
(**a**) Diagram of the fabrication process of PBCN; (**b**) WCAs of prepared nanofibrous membranes; (**c**) SEM image of PBCN nanofibrous membrane; (**d**) N_2_ adsorption–desorption isotherms of nanofibrous membranes (pore size distributions are shown in the insert); (**e**) photocatalytic degradation performance for different pollutants. Reprinted with permission from Ref. [150], Copyright 2022, Elsevier.

In addition to porous structure, the hydrophilicity of nanofiber material facilitates contact with a water medium, which is beneficial to its application in wastewater treatment. Xu et al. [150] took polyether sulfone and polyvinyl pyrrolidone (PVP) blends as raw materials and added boron-doped and nitrogen-lacking graphite carbon nitride for electrospinning (Figure 9a). Then, PVP was removed by simple washing to obtain highly porous and super-hydrophilic nanofiber membranes (PBCN). PVP, as a sacrificial pore-forming agent, built interlinked mesoporous channels inside the fiber (Figure 9c,d) and promoted the diffusion of pollutant molecules; it also exposed more photocatalytic sites and accelerated photocatalytic degradation. On the other hand, the introduction of PVP made the composite nanofiber membranes more hydrophilic (Figure 9b). The composite film showed a high removal rate of MB and excellent performances of recycling, regeneration, and repetition (Figure 9e). The hydrophilicity of porous nanofibers can be increased by creating hydrophilic pores or channels in them. This can accelerate mass transfer and further improve the photocatalytic performance of materials.

### 4.3. Energy Storage

The development of effective electrode materials is one of the critical elements in the advancement of clean, renewable energy technology. Electrospun fiber membranes with porous structures are considered as strong electrodes with excellent capacity and cycle stability due to their large surface area and abundant pores and are widely used in the energy storage field. The high porosity can enlarge the surface contact between the electrolyte and the electrode, offer rich charge transfer diffusion channels, and improve electron and ion transport dynamics. In addition, it may appropriately cache the variation in volume of an electrode material during the battery-charging and -discharging processes, ensure the structural integrity of a material, and further enhance the performance of electrochemical energy storage.

In the preparation of lithium ion battery electrodes, electrospun porous nanofibers have been successfully applied. Huang et al. [151] successfully synthesized porous carbon nanofibers through an easy-to-use electrospinning technique combined with an in situ pore formation process using a high-volatility solvent and a water bath collection device. These porous carbon nanofibers were used to encase sulfur to form porous carbon nanofiber/sulfur nanocomposites. Porous carbon nanofibers with large surface areas and highly porous structures are excellent substrates for sulfur limitation, according to electrochemical studies. The porous carbon nanofiber/sulfur nanocomposite had capacity retention rates of 80.1% and 68% after 50 and 100 cycles, respectively. Wang et al. [152] prepared three independent porous silicon@heteroatom-doped porous carbon fibers via coaxial electrospinning. The porous structure not only greatly alleviated the volume increase problem of silicon, but also provided abundant transmission and diffusion channels for lithium ions. After 100 cycles, the membrane’s capacity was 1145 mAh g^−1^, demonstrating an outstanding cycling performance. Consequently, the addition of porous structure is an approach that has promise for significantly enhancing the electrochemical properties of electrode materials.

**Figure 10:**
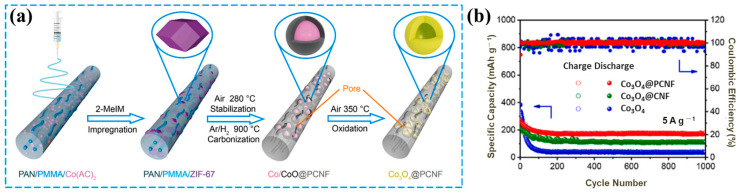
(**a**) Schematic illustration of the production of Co_3_O_4_@PCNF nanofibers; (**b**) cyclic performance of Co_3_O_4_@PCNF, Co_3_O_4_@CNF, and Co_3_O_4_. Reprinted with permission from Ref. [154], Copyright 2021, Elsevier.

As a commercial energy storage system, lithium ion batteries have achieved great success, but limited lithium resources and high cost limit further applications. Due to plentiful Na resources and extremely low cost, sodium ion batteries have recently replaced lithium ion batteries, which has aroused wide interest in researchers. Shan et al. [153] prepared N-doped graded carbon nanofibers (CZIF-8/PAN) using ZIF-8 particles as a template by electrospinning technology. Due to the addition of ZIF-8, the fiber film possessed a layered, porous structure after carbonization and acid treatment, which increased the surface contact area between the electrolyte and the electrode and improved the transfer speed of Na. Moreover, the doping of nitrogen atoms in the fibers also enhanced the active sites and electrical conductivity. After 600 cycles, the fiber membrane showed a remarkable cycling performance and a stable discharge capacity of 186.2 mAh g^–1^. Liao et al. [154] fabricated porous carbon nanofibers (Co_3_O_4_@PCNF) via electrospinning, in situ growth, and a two-step calcination process. Polyacrylonitrile (PAN)/poly(methyl methacrylate) (PMMA)/Co(Ac)_2_ electrospun fibers were immersed in 2-methylimidazole in situ to grow PAN/PMMA/ZIF-67 nanofibers. Then, using PMMA as a sacrificial template, mesoporous Co/CoO@PCNF was obtained after the first carbonization (900 °C), and finally, hollow Co_3_O_4_ was fixed in the carbon fiber matrix at the second oxidation pyrolysis (350 °C) to obtain Co_3_O_4_@CNF (Figure 10a). The creation of interpore structures in nanofibers was encouraged by the thermal degradation of PMMA, providing a multichannel route that improved ion and electron transport. In addition to significantly reducing the Na ion transport channel, the production of hollow Co_3_O_4_ nanoparticles could also effectively reduce the volume change during the process of sodification and desilication and could improve the structural stability. The composite membrane had an extremely high initial Coulomb efficiency (ICE of 91.6%), showing excellent sodium storage ability and a large reversible capacity after 1000 cycles (Figure 10b). The development of hollow-structured electrode materials for various energy storage devices is given a new direction by the successful production of porous nanofibers.

A few high-performance electrospun porous nanofibers can store sodium ions and lithium ions simultaneously. Zhu et al. [155] proposed a porous carbon nanofiber generated from Sn-MOF for the anodes of lithium and sodium ion batteries. A Sn-MOF organic framework was introduced to a PAN precursor solution for electrospinning. The obtained fiber membrane was then subjected to a carbothermic reduction reaction to obtain a layered porous fiber membrane (Sn@C@CNF). The porous structure accelerated the speed of the ion and electron transport rate and buffered the volume expansion of carbon inclusions of PAN fibers and the MOF skeleton, showing excellent cycling stability and electrochemical performance. The porous structure helped to increase the material’s capacity to store Li^+^ and Na^+^.

### 4.4. Biomedicine

Due to good biocompatibility, flexibility, biodegradability, and high porosity, electrospun porous nanofibers can increase drug delivery, antibacterial properties, and cell adhesion [156,157], showing a broad variety of application prospects in the clinical and medical fields. They are often used in drug controlled release [158,159], antibacterial [160], wound-dressing [161], and tissue-engineering [162,163] applications.

**Figure 11:**
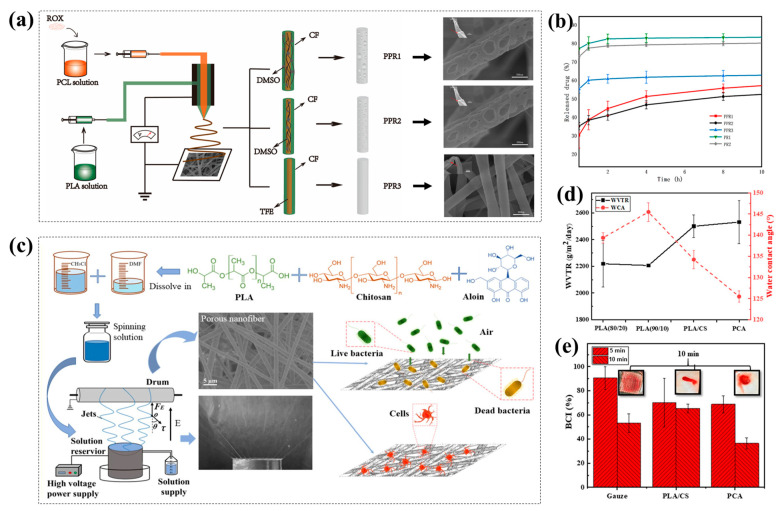
(**a**) Schematic diagram of preparation and SEM images of porous PCL/PLA nanofibers in different core–sheath solvent combinations by coaxial electrospinning method; (**b**) ROX release profiles of different nanofibers over 10 h. Reprinted with permission from Ref. [165], Copyright 2021, MDPI. (**c**) Illustration of the preparation of porous PLA/CS/aloin nanofibers (PCA); (**d**) water contact angles (WCAs) and water vapor transmission rates (WVTRs) of different nanofiber membranes; (**e**) in vitro blood coagulation test of nanofiber membranes. Reprinted with permission from Ref. [73], Copyright 2022, Springer.

Porous nanofibers are helpful for drug delivery and improved drug release, thus enhancing drug efficacy. Ramos et al. [164] prepared porous drug-loaded PCL nanofibers using high-humidity electrostatic spinning technology. The impacts of electrospinning processes (humidity and voltage), different solvent systems, and the presence of a model drug (chloramphenicol (CAM)) on the fiber porosity and drug release behavior were investigated. Porous PCL nanofibers with large pore size were produced in high-RH (65%) tetrahydrofuran (THF) and dimethyl sulfoxide (DMSO) solvent systems. Poreless drug-loaded PCL nanofibers had a large surface area because of macropores between the fibers. Although they had a fast drug release effect in the early stage, most drugs were wrapped in the fiber membrane, and only a small part of the drugs (about 20%) located on the fiber surface were released. Compared with nonporous fibers, porous nanofibers could release twice the amount of drug because of the pores on the surface of the fiber, exhibiting a better drug release performance. Therefore, the porous structure of porous electrospinning nanofibers can promote drug solubility and regulate the release rate of drugs. Coaxial electrospinning technology combined with NIPS was used by Chen et al. [165] to fabricate drug-loaded porous PCL/PLA nanofibers (Figure 11a). The impacts of various core–sheath solvent combinations on the microstructures of coaxial porous fibers were studied. The interaction of NIPS and BFs during electrospinning was primarily responsible for the creation of porous structures. Coaxial porous fibers, as opposed to nonporous fibers, encouraged the sustained release of medicines, preventing their explosive release (Figure 11b). They also increased the dissolution of hydrophobic drugs, thus having greater application potential for the continuous release of medications.

Electrospun porous nanofibers also have potential applications in antibacterial and wound-healing applications. Chen et al. [160] fabricated a graded, porous cellulose acetate (CA) nanofiber membrane covering thymol (THY) and β-cyclodextrin (β-CD) by electrospinning. The antibacterial activity of the porous fiber membrane against Staphylococcus aureus in vitro was studied. The composite membrane showed the highest porosity (86.0%) and high drug loading efficiency. The porous structure also increased the drug release time and showed more potent and long-lasting antibacterial properties against Staphylococcus aureus. The bacterial survival rate was 0.09% after 48 h. The porous nanofiber also had good cellular compatibility due to its high porosity and hierarchical porous structure, which provided a permeable network structure for cell proliferation and regulated the diffusion of metabolites and oxygen transport. The porous structure could also improve protein adhesion and promote cell adhesion, diffusion, and proliferation. This intriguing study expanded the potential use of porous nanofiber membranes as a novel material for wound healing. Yin et al. [73] added chitosan (CS) and aloin into a polylactic acid (PLA) solution and successfully prepared batch of porous PLA/CS/aloin nanofibers (PCA) for wound dressing using a self-made electrospinning device (Figure 11c). The introduction of CS and aloin improved the hydrophobic property of PCL (Figure 11d), which might lessen the dressing’s adherence to the wound and avoid harm from dressing removal. Meanwhile, the porous structure of PCA with high porosity could also facilitate water transport, and the porous fiber membrane had good hydrophobicity and a high swelling rate (Figure 11e). The combination of PLA and CS made the fiber membrane have good tensile strength. The positive CS also accelerated the aggregation of negatively charged platelets, and the porous membrane showed good in vitro coagulation ability (Figure 11f). The addition of aloin significantly increased the antimicrobial properties against Staphylococcus aureus and Escherichia coli, and the antibacterial rate reached 99.9%. Additionally, the porous structure gave the cells more anchorage points and strengthened their attachment to the matrix, thus further accelerating wound healing.

In addition, electrospun porous nanofibers have also attracted great attention in tissue engineering. Liu et al. [166] prepared a porous poly (3-hydroxybutyrate co-4-hydroxybutyrate) (P34HB) scaffold covered with lecithin that self-assembled on the surface of a scaffold by electrospinning technology. Porous scaffolds provided mesenchymal stem cells from bone marrow protection and sufficient three-dimensional space for cell proliferation. Combined with lecithin, they synergically promoted osteogenesis and regeneration. The obtained scaffold had good hydrophilicity and biocompatibility, and the interlinked pores were able to transport oxygen and nutrients to the cells, promoting cell attachment and growth. One of the best techniques for creating the macropores necessary for cell migration and tissue regeneration in tissue engineering is freeze-drying. Ahmed et al. [167] produced PCL imitation cotton fiber (PCLCLF) by electrospinning and blowing technology, which was then mechanically compressed into scaffolds, permeated with a collagen (COL) solution of water gel, and lyophilized to obtain high-volume and extremely porous PCL/COL scaffolds. The scaffolds showed a high porous structure, as well as high water absorption capacity and cutting ability, which could be used for the regeneration of large-volume tissue defects that were not load-bearing.

### 4.5. Other Applications

In addition to the above fields, electrospun nanofibers’ porous structures give them special structural and functional merits in food packaging, sensors, sound and wave absorption, heat insulation, flame retardant applications, and so on.

**Figure 12:**
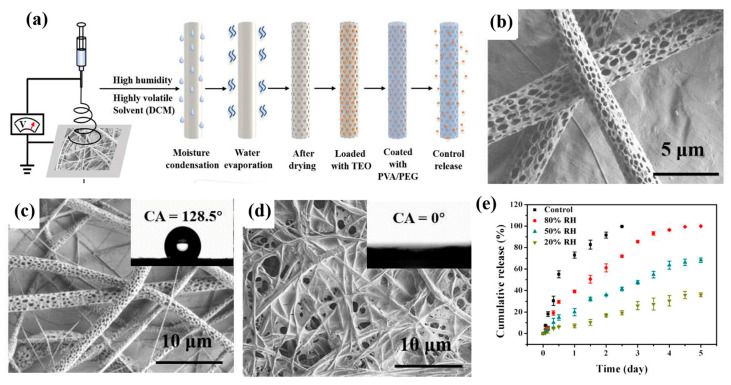
(**a**) Preparation process of PLA/TEO/PVA/PEG composite membrane under high-humidity conditions; (**b**) SEM photo of porous nanofiber membrane; (**c**,**d**) water contact angles of pure PLA and hydrophilic PLA/TEO/PVA/PEG nanofibers; (**e**) release behaviors of thyme essential oil (TEO) (at 20%, 50%, and 80% relative humidity). Reprinted with permission from Ref. [168], Copyright 2021, Elsevier.

Bioactive substances can be transported using electrospun porous nanofibers, which are useful for food packaging. Using electrospun porous polylactic acid (PLA) nanofibers as a carrier, Min et al. [168] fabricated a new antibacterial-packaged PLA/ thyme essential oil (TEO)/PVA/PEG composite film that was loaded with thyme essential oil (TEO) and hydrophilic polyvinyl alcohol(PVA)/polyethylene glycol (PEG) by a soaking method (Figure 12a,b). The addition of PVA/PEG improved the fiber membrane’s hydrophilicity (Figure 12c,d). The porous structure of the PLA fiber provided more space for TEO, which enabled TEO to be wrapped into the pores and improved the loading ability of TEO. By adjusting the ambient humidity (20–80% relative humidity), the release rate of TEO in the composite membrane gradually increased (Figure 12e), showing excellent antibacterial activity, which offered a novel approach to the design and development of food active-packaging materials.

**Figure 13:**
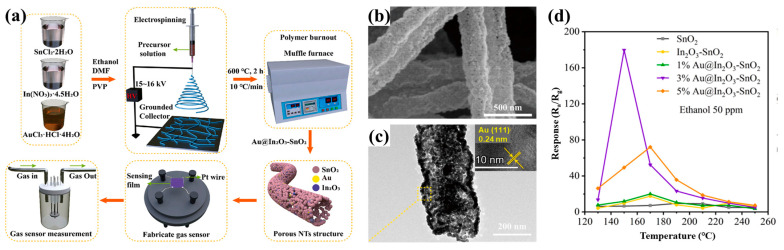
(**a**) Schematic diagram of the designed gas sensor of the porous Au@In_2_O_3_-SnO_2_ nanotubes; (**b**–**c**) SEM and TEM images of 3% Au@In_2_O_3_-SnO_2_; (**d**) response variation to ethanol of different sensors at different temperatures. Reprinted with permission from Ref. [172], Copyright 2023, Elsevier.

Research on the preparation of sensors with huge specific surface areas, high porosity, high sensitivity, and high selectivity by electrospinning technology has been widely reported [169,170]. The addition to porous structure, the electrostatic spinning of nanofibers’ surfaces can enhance molecule diffusion and improve the sensing performance. Furthermore, by regulating pore size, selectivity can be attained. Cai et al. [171] developed porous TiO_2_ nanofibers doped with Co_3_O_4_ using electrospinning and hydrothermal processes to enhance the gas-sensitive properties of acetone. The fiber’s specific surface area was significantly increased by the porous structure, which also increased the number of porous channels available for acetone gas absorption. In addition, porous TiO_2_ nanofibers modified with Co_3_O_4_ showed superior acetone gas selectivity and long-term stability. The porous structure and p–n junction formed at the interface of TiO_2_ and Co_3_O_4_ significantly enhance the sensing performance of nanofibers. Chen et al. [172] prepared Au-decorated porous SnO_2_-doped nanotubes (Au@In_2_O_3_-SnO_2_) by electrospinning and calcination at 600 °C (Figure 13a). The porous structure (Figure 13b,c) and high surface area offered additional reaction sites, which was conducive to the adsorption and desorption of the target gas. It also sped up the sensing reaction that occurred between oxygen and ethanol. The 3% Au@In_2_O_3_-SnO_2_ sensor showed a high Ra/Rg value of 179.62 for 50 ppm ethanol (Figure 13d). The large amount of pores, more active sites, Au catalyst modification, and In^3+^-equivalent doping were thought to be the causes of the superior sensing properties. This work opened up more possibilities for creating different kinds of gas sensors.

**Figure 14:**
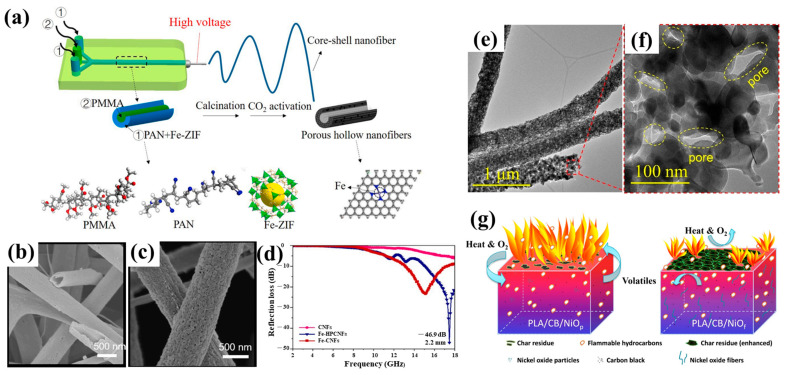
(**a**) Schematic of fabricating Fe-HPCNFs; (**b**,**c**) SEM photos of Fe-HPCNFs before and after liquid nitrogen cooling; (**d**) reflection loss curves for CNF, Fe-CNF, and Fe-HPCNF materials. Reprinted with permission from Ref. [175], Copyright 2022, Elsevier. (**e**,**f**) TEM and enlarged TEM images of porous NiO nanofibers; (**g**) the flame-retardant mechanism of PLA/CB/NiO_p_ and PLA/CB/NiO_f_ composites. Reprinted with permission from Ref. [178], Copyright 2020, Elsevier.

Both people and animals in the natural world are permanently affected by noise [173]. Electrospun porous fiber was proved to be a good sound-absorbing material because of its low weight, high porosity, low cost, wide range of sound absorption frequency, and strong sound absorption ability. Chao et al. [174] obtained macroporous silica nanofibers by electrospinning and high-temperature calcination. PVP, PS, and ethyl orthosilicate (TEOS) were used as a spinning solution for electrostatic spinning, and then the PVP and PS were removed by calcination at 500 °C. The great majority of the pores in the porous fiber were beneficial to attenuate sound energy and greatly enhanced the silica fiber’s ability to absorb sound. In the frequency range of 4.0–5.1 kHz, the porous silica nanofibers had a greater sound absorption coefficient than commercial sponges. In addition, electrospun porous nanofibers have great potential in absorbing electromagnetic waves. Sun et al. [175] prepared hollow porous carbon nanofibers (Fe-HPCNFs) by electrospinning a PAN precursor solution with additional Fe-ZIF, and then calcined it at a high temperature (Figure 14a–c). Compared with ordinary nanofibers, Fe-HPCNFs could absorb more EMWs and showed excellent electromagnetic wave absorption performance (Figure 14d). This was due to the fact that the existence of Fe-ZIF and the creation of hollow porous structures are crucial in controlling the impedance matching of fiber. The hollow porous structure had good impedance-matching characteristics, which further enhanced the attenuation of wave energy.

The various reflection effects of thermal radiation can be significantly improved by electrospun nanofibers because they have hierarchical porous structures and numerous solid–gas interfaces [176]. Convective heat transfer is further lessened by the air in pores, which aids in lowering a material’s thermal conductivity. As a result, it is possible to attain better heat insulation and flame-retardant properties. Wang et al. [177] used PVP, tetraethyl orthosilicate (TEOS), and titanium butoxide (TBT) as a precursor solution for electrospinning and calcined the obtained fiber film to obtain a SiO_2_-TiO_2_ sponge with a micro-meso-macroporous layered porous structure. The pores in the sponge were distributed in a gradient from the center of the fiber to the wall of the fiber and were connected with each other, showing high porosity, low packing density, and good compressibility. The sponge still maintained a low temperature after being treated above the flame of a 500 °C alcohol lamp for 30 min. A porous structure could be included into the fiber to increase the material’s flame retardancy. Zhang et al. [178] prepared nickel oxide (NiO) porous nanofibers using electrospinning and pyrolysis techniques (Figure 14e,f). The mesoporous structure promoted the interaction between NiO_f_ and a polylactic acid matrix (Figure 14g), and the mechanical test showed excellent dispersion and better load transfer capability. Compared with commercially available NiO particles, the prepared multiresistant NiO fibers had better flame-retardant properties. In the future, combinations of this porous structure with electrospun multichamber structures (such as three-layer parallel and chimeric Janus structures [179,180,181]) could result in more functional nanoproducts.

## 5. Conclusions and Perspectives

The usage of electrospinning technology, one of the most popular techniques for preparing nanofibers, is widespread in various fields due to its advantages, such as ease of use, controllable process and fiber size, and repeatability. The specific surface area of a fiber is increased even more by the addition of a porous structure, which also considerably enhances a material’s performance. The preparation methods of porous nanofiber membranes by electrospinning, including the template method and the phase separation method, were reviewed in this paper. In the template method, a certain template is selected, and the template is removed by a post-processing method to obtain porous nanofibers. In the phase separation technique, porous nanofibers can be produced directly by controlling the parameters of electrospinning (such as humidity, temperature, etc.) and selecting suitable polymers and solvents. A porous structure not only greatly increases the surface areas of fibers and provides a large adsorption capacity but also can serve as a diffusion channel to provide a large number of reaction sites and speed up the diffusion and transfer of substances. By designing porous structures at different scales or loading functional particles on porous nanofibers, the performances of materials can be further improved, and materials with different functions can be prepared, showing great application prospects in air purification, water treatment, biomedicine, energy storage, food packaging, and so on.

However, there are still some challenges: (1) Because interior structure greatly affects the properties of materials, it is important to optimize the design of electrospun porous nanofiber structures and to innovate more suitable methods for building highly ordered porous structures inside them. (2) The mechanical properties of electrospun nanofibers are unavoidably impacted by their porous structures. The interactions between mechanical properties and porous structures must be balanced. Therefore, higher requirements should be put forward for the properties and structural stability of porous nanofibers. (3) Due to their high cost and low production efficiency, electrospun nanofibers are still only prepared and used in academic research fields. More attention should be paid to the formation mechanisms of porous nanofibers to develop production methods and equipment suitable for industrial applications. Electrospun porous nanofibers have become a research hotspot, so with the progress and development of electrospinning technology and the unremitting efforts of scientists and researchers, more excellent performances of electrospun porous nanofibers can be developed. The expansion of their applications can be further strengthened along with the fast developments in modern science and technology, such as encapsulating new functional ingredients [182,183,184] and additives [185,186], exploring new types of filament-forming polymeric matrices [187,188], being introduced to popular life-improving applications [189,190,191,192,193,194], taking advantage of new strategies of synthesizing materials [195,196], and drawing support from traditional techniques for production on a large scale [197,198].

## Figures and Tables

**Table 1:** Comparison of different phase separation mechanisms, formation conditions, and pores.

Type	Mechanism	Polymer	Solvent	Humidity	Pore Structure	Pore Size (nm)	Porosity	Ref.
VIPS	Water molecules are mixed with a low-volatile solvent, resulting in phase separation.	Hydrophobic polymer	Single, low-volatile, miscible with water	High	Surface and internal, elliptical	Large, 50–300	Reached 92%	[118,119]
TIPS	Significant temperature difference between fibers and the surrounding environment results in phase separation.	Not required	Single, low-volatile	Not required	Surface	Small, 2–50	Lower than other pore-forming mechanisms	[113,120]
NIPS	Volatility difference between the solvent and nonsolvent results in phase separation.	Not required	High-volatile solvent and low-volatile nonsolvent	Not required	Surface and internal, elliptical	Small, 20–100	Less than 80%	[55,121]

## Data Availability

The data supporting the findings of this manuscript are available from the corresponding authors upon reasonable request.
